# Age-, sex-, and maturity-associated variation in the phase angle after adjusting for size in adolescents

**DOI:** 10.3389/fnut.2022.939714

**Published:** 2022-08-01

**Authors:** Anderson M. de Moraes, Ricardo T. Quinaud, Giovana O. C. Ferreira, Ahlan B. Lima, Humberto M. Carvalho, Gil Guerra-Júnior

**Affiliations:** ^1^Department of Physical Education, School of Sports, Pontifical Catholic University of Campinas, São Paulo, Brazil; ^2^Department of Physical Education, University of Extreme South of Santa Catarina, Criciúma, Santa Catarina, Brazil; ^3^Laboratory of Growth and Development, Center for Investigation in Pediatrics, Department of Pediatrics, School of Medical Sciences, University of Campinas, São Paulo, Brazil

**Keywords:** pediatric populations, body mass, body composition, youth, Bayesian methods, multilevel modeling

## Abstract

**Background:**

Applied research using the phase angle (PhA) in children and adolescents has increased notably. Using multilevel modeling in a fully Bayesian framework, we examined the relationships between PhA, age, sex, biological maturity status, and body size in 10–16-year-old adolescents.

**Methods:**

The sample comprised 519 adolescents (women, *n* = 241; men, *n* = 278) from Campinas, São Paulo, Brazil. Biological maturity status was assessed with self-examination of pubertal development for sexual maturity and maturity offset protocol to estimate age at peak height velocity (PHV) for somatic maturity status. Stature and body mass were measured by anthropometry. Phase angle was calculated based on raw resistance and reactance values (50 kHz frequency) obtained by bioelectrical impedance with the foot-to-hand technology.

**Results:**

The multilevel regression analysis revealed that boys had significantly higher values of phase angle than girls, adjusting for age group and sexual maturity status. Overall, older and more mature adolescents had higher values of phase angle. When considering aligning variation in the phase angle by distance to estimated PHV (maturity offset), there was a higher association between the phase angle and time before and after predicted age at PHV for boys (*r* = 0.31, 90% CI: 0.23 to 0.39) than girls (*r* = 0.2, 90% CI: 0.11 to 0.28). When including body mass in the multilevel models, corresponding changes in the overall body mass mediate most of the influence of the maturity status and age group on the phase angle.

**Conclusion:**

The present study demonstrated that the variability in phase angle is related to inter-individual variation in sex, age, and maturity status, as well as differences in body size. Research with adolescents considering phase angle should use multilevel modeling with standardized parameters as default to adjust for the concurrent influence of sex, age, maturity status, and body size.

## Introduction

Bioelectrical bioimpedance analysis (BIA) has been an attractive method to assess body composition (1). The BIA provides an easy-to-handle, non-invasive, portable method with good reproducibility, which is viable for clinical practice and epidemiological studies (2–5). BIA measures the values of resistance (*R*) and reactance (*Xc*) of a current as it passes through tissues of the body measured (6, 7). *R* represents the opposition offered by the body to the flow of an alternating electrical current and is inversely related to the water and electrolyte content of tissues (5). *Xc* represents the resistive effect produced by the tissue interferences and cell membranes (5, 6). Body composition has indirectly been estimated using prediction equations from R and Xc measurements derived with BIA (7). However, age-, ethnic-, sex- or clinical-conditions-associated variations in body shape, relations between the trunk and leg lengths, and hydration levels limit the validity of the equations (6, 7), allowing for the validity of the modeling assumptions of the prediction equations.

Information about body fluids distribution among intracellular and extracellular compartments and tissue integrity can be obtained from raw BIA measurements, *R* and *Xc* (2, 6, 8). The values of *R* and *Xc* of a current as it passes through tissues of the body by BIA can be used to calculate the phase angle, PhA (2, 9). Since bioelectric impedance (*Z*) results from the two vectors representing R and Xc, PhA is the angle between *Z* and *R* (10, 11). PhA is influenced by the body quantity of cells, with respective cell membranes, cell membrane integrity, related permeability, and the amounts of intracellular and extracellular fluids (1, 7). Consequently, factors such as age, sex, body dimensions and compositions, level of physical activity, or fluid status should be considered when interpreting PhA (1, 2, 12, 13). PhA is a relevant health parameter in clinical use as it reflects the body fluid distribution among intracellular and extracellular compartments (2, 8). Furthermore, PhA can indicate the nutritional status of different populations and malnourished children, healthy children, and adolescents (14–16).

Recently, the interest in applied research interpreting PhA in children and adolescents, particularly in youth sports, has increased notably (14–19). The PhA values should increase with adolescence and should be more pronounced in boys than girls (10, 13). During the pubertal growth spurt, gains in fat-free mass are higher in boys, while relative fatness, i.e., fat mass as a percentage of body mass, tends to decline in boys but increase in girls (20). Phase angle increases with age in adolescence, particularly in boys, due to a increase in reactance which parallels the gains of fat-free mass (particularly muscle mass) and a decrease in resistance due to the increasing proportion of body water at the expense of reduction of relative fatness.

Substantial body dimensions and composition changes mark pubertal growth, and sexual dimorphism becomes apparent (21). However, biological maturity-associated variation between individuals in body size and composition is considerable during pubertal years (22). Moreover, within a small chronological age range during adolescence, differences in maturity status may be significant within and between boys and girls. Hence, pubertal growth changes and maturity-associated variation in body dimensions and composition likely influence the interpretation of PhA, particularly among adolescents around peak height velocity (PHV) age (23). However, data considering the influence of maturity status on PhA is limited and mostly based on samples of young athletes (24–28). Overall, the interpretation of maturity-related variation aligned with adolescents' chronological age, sex, and body size merits further study.

Interpretations of physiological outcomes, particularly considering children and adolescents, need to consider cross-classified nesting within and between groups (e.g., sex, maturity status, age group), which often requires coping with an imbalance in sample size and heterogeneity among individuals. Therefore, multilevel models should be used as default, as they allow and explicitly model the data structure by allowing for residual components at each level in the hierarchy or cluster (29, 30). Nevertheless, Traditional single-level regressions continue to be used to deal with data in pediatric physiology, which is a concern, particularly, in settings with a low group-level variation where multiple comparisons exist (31).

In the present study, using Bayesian multilevel modeling, we examined the age-, sex-and maturity-associated variation in PhA adjusting for the influence of body size in adolescents aged 10 to 16.

## Materials and methods

### Study design and sample

This study used a cross-sectional design on 519 adolescents (girls, *n* = 241; boys, *n* = 278) from Campinas, São Paulo, Brazil. Participants in this study were adolescents enrolled adequately at the local school, detaining a regular frequency of physical education. In addition, the study did not consider children with physical disabilities (permanent or temporary), children impeding participation in any of the procedures, or those using prescribed medicine. For each participant, all the measurements were obtained in the morning, after an overnight regular fast (8 h), refraining from vigorous exercise for at least 15 h, avoiding caffeine and alcohol during the preceding 24 h, and consuming a normal evening meal the night before.

The Ethics Committee of the Pontifical Catholic University of Campinas (CAAE: 79625817.6.0000.5481) approved the research. All procedures followed Resolution No.466 of 2012 of the National Health Council of the Ministry of Health of Brazil and were conducted following the Declaration of Helsinki. Participants and their parents or legal guardians provided informed written consent.

### Age and anthropometry

Chronologic age was calculated and recorded to the nearest 0.1 years by subtracting the birth date from testing. The participants were categorized by age group as follows: 10–11 years old (10 to 11.9 years); 12–13 years old (12 to 13.9 years); and 14–15 years old (14.to 15.9 years). Stature was measured with a vertical portable stadiometer (Sanny, SBC, SP, Brazil) to the nearest 0.1 cm. Body mass was measured with a calibrated portable balance (Sanny Digital Glass 200 Control, SBC, SP, Brazil) to the nearest 0.05 kg. Technical errors of measurement were 0.29 cm for stature and 0.51 kg for body mass, based on replicated measurements of 20 participants.

### Maturity status

We estimated the maturity status using two approaches: (i) sexual maturation, using self-examination of pubertal development, and (ii) somatic maturation, using estimations with the maturity offset and age at PHV using sex-specific equations (32).

#### Sexual maturation

Before the self-physical examination, the participants were provided with a standardized series of realistic color images with an explanatory text to individually assess their pubertal development (32), following the sexual maturity stage criteria described by Tanner (33). For example, in girls, breast development was classified from 1 (pre-puberty) to 5 (mature), and stage 2 (appearance of the buttoned breast) marks the beginning of pubertal development. In boys, genital development was classified from 1 (pre-puberty) to 5 (mature); stage 2 marks puberty onset. Participants were asked to read brief descriptions of each stage and check the box on the image that best represents the development component. All assessments were carried out in a private room. Participants were grouped as pre-puberty (classified as 1), early puberty (classified as 2), mid-puberty (classified as 3), late puberty (classified as 4), and mature (classified as 5).

#### Somatic maturation

We used the simplified versions of the gender-specific maturity offset protocol (32) to determine participants' maturity status. The offset equations estimate time before or after PHV based on their chronological age and stature. Thus, negative values indicate the time before PHV, and positive values indicate the time after PHV.

### Phase angle

PhA was assessed using a single frequency (50 kHz) BIA device, model Quantum II (RJL Systems, Detroit, MI, USA). All adolescents were instructed to remove all objects containing metal before taking the BIA measurement. Next, participants were laid barefoot, in a supine position, with the legs abducted at a 45° angle, arms far from the trunk, and hands pronated on a table isolated from electrical conductors. After 5 min of resting, the participants' skin was cleaned with alcohol, and two electrodes were placed on the surface of the right hand and two others on the surface of the right foot, according to the recommended protocol (9). The evaluation lasted approximately 1 min.

Bioelectrical bioimpedance analysis provided the value of *R* and *Xc* in ohms (Ω), and, from these variables, PhA was calculated using the following published equation (9):


(1) $$\begin{array}{l} PhA=arctn\;\left(Xc/R\right)\times \;\left(180/\pi \right) \end{array}$$

Based on replicated measurements of 23 participants, the technical errors of measurement were 3.54 and 0.49 Ω for *R* and *Xc*, respectively. Corresponding coefficients of variation were 0.35 and 0.33% for *R* and *Xc*, respectively.

### Statistical analysis

We used a fully Bayesian approach in our analysis. By fitting the multilevel models within a Bayesian framework (34), the parameters are treated as random variables combining both prior distribution information and sample data to estimate a (posterior) probability distribution that reflects the uncertainty associated with how well they are known based on the data (35, 36). Thus, Bayesian methods allow a direct probabilistic interpretation of CIs (also referred to as confidence or compatibility intervals) and posterior probabilities, relevant in applied human biology research, where the interest frequently lies in estimating small effects.

Our estimations were based on the Bayesian multilevel models considering the variation in PhA, adjusting for cross-classified nesting by age group, sex, and maturity status among young Brazilian adolescents. We standardized (z-score) all the outcomes for interpretative convenience and computational efficiency. Given the limitations in the agreement between maturity indicators, we explored the influence of maturity status by considering the sexual maturity status as a discrete variable with five levels (pre-puberty, early puberty, mid-puberty, late puberty, and mature) and the somatic maturity status using the maturity offset as a continuous variable.

To model the influence of age group, sex, and sexual maturity status on PhA, we used varying-intercept models where each participant's outcome (intercept) was estimated as a function of his/her age group, sex, and estimated sexual maturity status (model 1). Hence, for individual *i*, we used indexes *a, s*, and *m* for age group, sex, and sexual maturity status, respectively. The group-level effect terms (also referred to as random effects) and the data-level terms (also referred to as level-1 residuals) were drawn from normal distributions with variances to be estimated from the data:


(2) $$\begin{array}{lll} y_{i}=\beta_{0} & + & \alpha_{a\left[i\right]}^{age\;group}+\alpha_{s\left[i\right]}^{sex}+\alpha_{m\left[i\right]}^{maturity\;status}+\epsilon_{i} \end{array}$$

(3) $$\begin{array}{l} \alpha_{a\left[i\right]}^{age\;group}\;\sim \;N\left(0,\;\sigma_{age\;group}^{2}\right),\;for\;a=1,2,3. \end{array}$$

(4) $$\begin{array}{l} \alpha_{s\left[i\right]}^{sex}\;\sim \;N\left(0,\;\sigma_{sex}^{2}\right),\;for\;s=1,2. \end{array}$$

(5) $$\begin{array}{l} \alpha_{m\left[i\right]}^{maturity\;status}\;\sim \;N\left(0,\;\sigma_{maturity\;status}^{2}\right),\;for\;m=1,2,3. \end{array}$$

(6) $$\begin{array}{l} \epsilon_{i}\;\sim \;N\left(0,\;\sigma_{y_{i}}^{2}\right) \end{array}$$

We replicated the model 1 structure, adding body mass as a populations level effect (model 2):


(7) $$\begin{array}{l} y_{i}=\beta_{0}+\beta_{1}^{body\;mass}+\alpha_{a[i]}^{age\;group}+\alpha_{s[i]}^{sex}\\ \;+\alpha_{m[i]}^{maturity\;status}+\;\epsilon_{i} \end{array}$$

(8) $$\begin{array}{l} \alpha_{a\left[i\right]}^{age\;group}\;\sim \;N\left(0,\;\sigma_{age\;group}^{2}\right),\;for\;a\;=\;1,2,3. \end{array}$$

(9) $$\begin{array}{l} \alpha_{s\left[i\right]}^{sex}\;\sim \;N\left(0,\;\sigma_{sex}^{2}\right),\;for\;s\;=\;1,2. \end{array}$$

(10) $$\begin{array}{l} \alpha_{m\left[i\right]}^{maturity\;status}\;\sim \;N\left(0,\;\sigma_{maturity\;status}^{2}\right),\;for\;m\;=\;1,2,3. \end{array}$$

(11) $$\begin{array}{l} \epsilon_{i}\;\sim \;N\left(0,\;\sigma_{y_{i}}^{2}\right) \end{array}$$

Considering the somatic maturity status, we used a varying-intercept and a varying-slope model to model PhA as a function of his/her estimated maturity offset, age group, and sex (model 3). We allowed for individuals' maturity offset to vary by sex (varying slope):


(12) $$\begin{array}{lll} y_{i}=\beta_{0} & + & \beta_{s\left[i\right]}^{maturity\;offset}+\alpha_{s\left[i\right]}^{sex}+\alpha_{a\left[i\right]}^{age\;group}+\epsilon_{i} \end{array}$$

(13) $$\left[\begin{array}{c} \beta_{0}\\ \beta_{s[i]}^{maturity\;offset} \end{array}\right]\;\sim \;MVNormal\left(\left[\begin{array}{c} \beta_{0}\\ \beta^{maturity\;offset} \end{array}\right],\Sigma \right)$$

(14) $$\Sigma \;\sim \;\left[\begin{array}{cc} \sigma_{\beta_{0}} & 0\\ 0 & \sigma_{\beta^{maturity\;offset}} \end{array}\right]R\left[\begin{array}{cc} \sigma_{\beta_{0}} & 0\\ 0 & \sigma_{\beta^{maturity\;offset}} \end{array}\right]$$

(15) $$\begin{array}{l} \alpha_{s\left[i\right]}^{sex}\;\sim \;N\left(0,\;\sigma_{sex}^{2}\right),\;for\;s=1,2. \end{array}$$

(16) $$\begin{array}{l} \alpha_{a\left[i\right]}^{age\;group}\;\sim \;N\left(0,\;\sigma_{age\;group}^{2}\right),\;for\;a=1,2,3. \end{array}$$

(17) $$\begin{array}{l} \epsilon_{i}\;\sim \;N\left(0,\;\sigma_{y_{i}}^{2}\right) \end{array}$$

Again, we added body mass as a population-level effect to the model, but in this case, a varying-intercept model was used (model 4):


(18) $$\begin{array}{l} y_{i}=\beta_{0}+\beta_{1}^{maturity\;offset}+\beta_{2}^{body\;mass}+\alpha_{s[i]}^{sex}\\ \;+\alpha_{a[i]}^{age\;group}+\epsilon_{i} \end{array}$$

(19) $$\begin{array}{l} \alpha_{s\left[i\right]}^{sex}\;\sim \;N\left(0,\;\sigma_{sex}^{2}\right),\;for\;s=1,2. \end{array}$$

(20) $$\begin{array}{l} \alpha_{a\left[i\right]}^{age\;group}\;\sim \;N\left(0,\;\sigma_{age\;group}^{2}\right),\;for\;a=1,2,3. \end{array}$$

(21) $$\begin{array}{l} \epsilon_{i}\;\sim \;N\left(0,\;\sigma_{y_{i}}^{2}\right) \end{array}$$

We used weakly informative priors to regularize our estimates, a normal prior (0,5) for the intercept (population-level parameter, also referred to as fixed effect) and a normal prior (0,1) for group-level parameters. For the data-level residuals (ϵ_*i*_), we used the “brms” default prior, Student-*t* (3, 0, 2.5) (37). Considering the outcome standardization, by using a normal (0,1) prior for the parameters, we state that the group-level estimates are unlikely to be greater than one standard deviation. To check whether the models successfully partitioned the influence of body mass (when included in the model), we inspected the residual plots against the body mass to check the homoscedasticity of the residuals. We ran four chains for 2,000 iterations with a warm-up length of 1,000 iterations in each model. The convergence of Markov chains was inspected with trace plots. We used posterior predictive checks to be confident in our models and estimations (34). We fitted the models in R (38) using the “brms” package (37), which calls Stan (39).

## Results

The distribution of stages of pubic hair within age groups by sex is given in Table 1. The girls aged 10–11 were mainly distributed in early puberty, mid-puberty, and late puberty. Boys aged 10–11 years old were mainly classified as mid-puberty. Both girls and boys aged 12–13 were mainly classified as mid-puberty and late puberty, and 20 out of 104 girls in the age group were classified as mature. Girls aged 14–15 were mainly in late puberty (PH4) or mature. Boys aged 14–15 years were mainly in late puberty (~47%), but about 32% were in mid-puberty, and 20% were mature.

**Table 1:** Distribution of stages of pubic hair (PH) in the sample of adolescents by sex and age group.

	**PH1**	**PH2**	**PH3**	**PH4**	**PH5**
*Female*					
10–11 years	2	20	33	26	1
12–13 years	0	2	21	71	20
14–15 years	0	1	2	31	11
*Male*					
10–11 years	3	11	56	9	1
12–13 years	0	4	42	80	12
14–15 years	0	1	19	28	12

Estimated maturity offset in the sample of adolescents by sex and age group is summarized in Table 2. On average, offset values were higher for girls across all age groups. In addition, values of offset increased as participants were older. However, the range of values in each age group was extensive (about 2 years), indicating significant variations between participants in the somatic maturity status within age groups.

**Table 2:** Mean, standard deviation and range of maturity offset in the sample of adolescents by sex and age group.

	**Mean**	**Standard deviation**	**Range**
*Female*			
10–11 years	^−0.77^	^0.55^	−2.04 to 0.51
12–13 years	^0.77^	0.70	−0.60 to 2.36
14–15 years	^2.09^	0.49	1.13 to 3.02
*Male*			
10–11 years	−2.06	0.48	−3.21 to −0.97
12–13 years	−0.62	0.63	−1.86 to 0.83
14–15 years	0.81	0.56	−0.39 to 2.60

The characteristics of the sample by sex are summarized in Table 3.

**Table 3:** Descriptive statistics (mean and standard deviation) for adolescents by sex.

	**Female (*****n*** = **22)**	**Male (*****n*** = **35)**
Chronological age (yrs)	12.7 (1.3)	12.8 (1.3)
Maturity offset (yrs)	0.49 (1.20)	−0.72 (1.17)
Stature (cm)	152.9 (9.0)	156.2 (11.0)
Body mass (kg)	48.2 (11.5)	51.4 (14.6)
Phase angle (degree)	5.50 (0.70)	5.88 (0.77)

**Figure 1:**
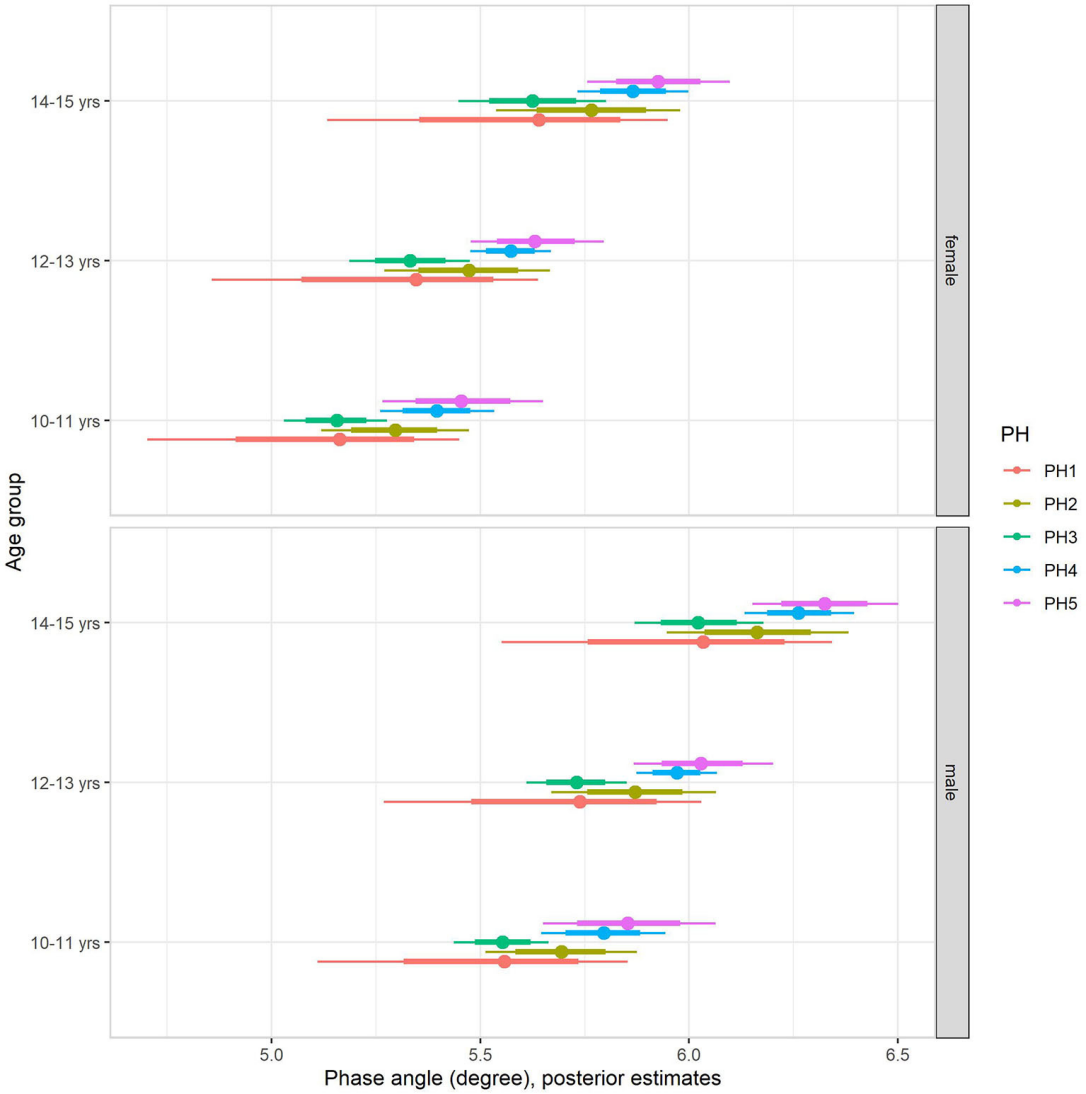
Posterior estimations (67 and 90% CIs) for the phase angle by age group and sexual maturity among Brazilian girls and boys.

The multilevel regression models examining variation in PhA associated with gender, age group, and sexual maturity stage are summarized in model 1 of Table 4. Note that outcomes were standardized in the models, and the table summaries are presented on a z-score scale. Back transformed posterior estimates of adolescents' PhA are given in Figure 1, contrasting boys and girls by sexual maturity stage within each age group. Overall, older and more mature adolescents had higher values of PhA. The results of model 1 show substantial variation by sex. Boys had higher values than girls, adjusting for age group and sexual maturity stage (Figure 1). Also, the results showed an influence of age group and sexual maturity stage on PhA.

**Table 4:** Multilevel regression models posterior estimations and 90% credible intervals of variation in phase angle by sex, age group, stages of pubic hair (PH) (model 1), and adjusting for body mass (model 2) among adolescents.

	**Model 1** **(variation by** **sex, PH, and** **age group)**	**Model 2** **(variation by sex**, **PH, and age group**, **adjusted for** **body mass)**
Population-level effects (90% credible interval)		
Intercept	−0.03(−1.20 to 1.11)	0.01 (−1.01 to 1.04)
Body mass	-	0.34 (0.26 to 0.41)
Group level estimates (90% credible interval)		
Level 2, standard deviation		
Sex	0.73 (0.22 to 1.65)	0.67 (0.18 to 1.58)
PH	0.33 (0.10 to 0.75)	0.19 (0.02 to 0.49)
Age group	0.58 (0.19 to 1.31)	0.35 (0.06 to 0.98)
Level 1 standard deviation	0.92 (0.87 to 0.97)	0.88 (0.83 to 0.98)

All outcomes were standardized in the models.

**Figure 2:**
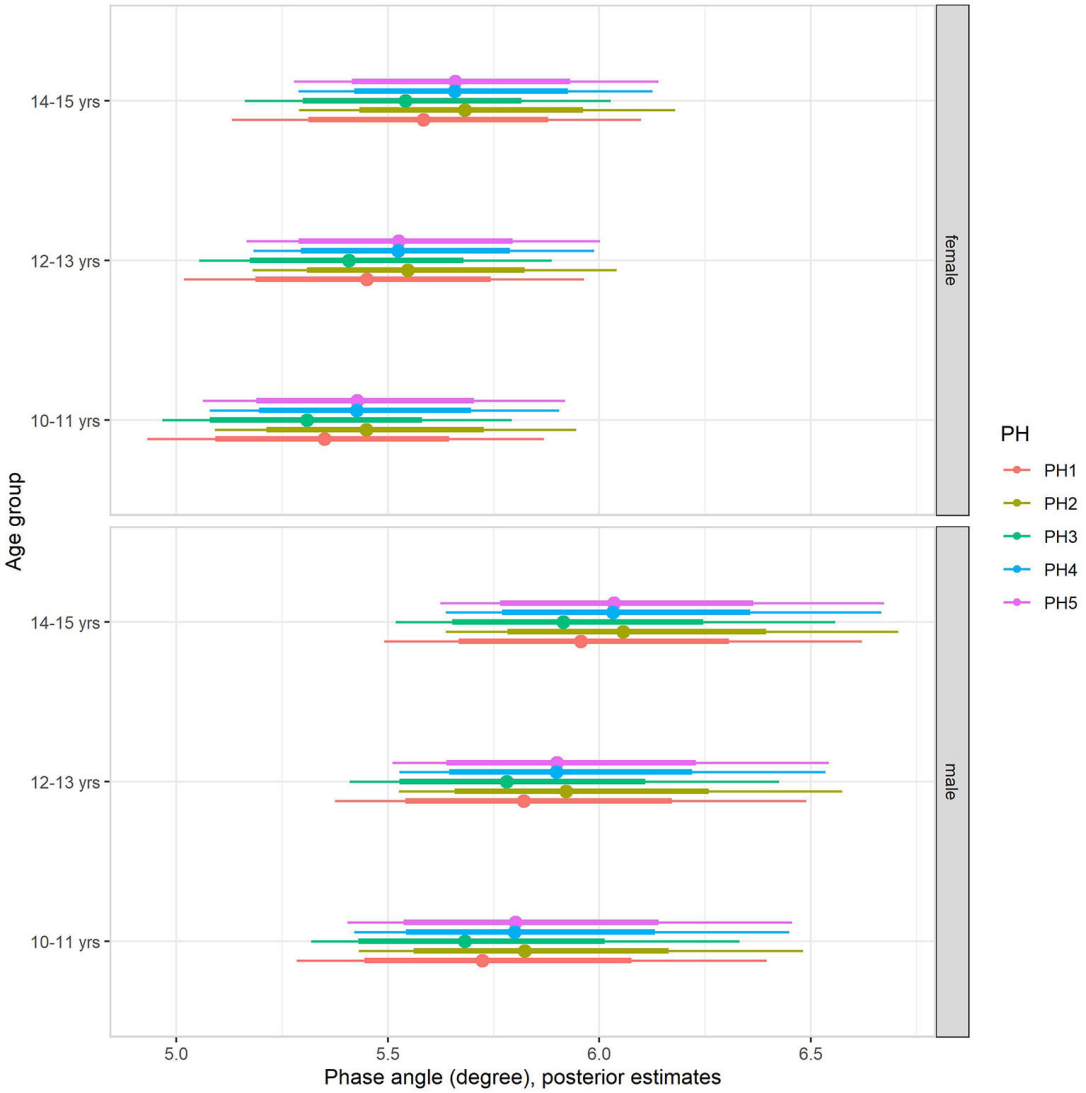
Posterior estimations after adjusting for body mass (67 and 90% CIs) for the phase angle by age group and sexual maturity among Brazilian girls and boys.

Back transformed posterior estimates of PhA adjusted for body mass, sex, age, and maturation are given in Figure 2, contrasting boys and girls by sexual maturity stage within each age group. When including body mass in the multilevel regression model (model 2, Table 4), it became clear that body mass substantially influenced PhA, independent of sex, age group, or sexual maturity stage. The sex-associated variation was attenuated but remained substantial (Figure 2). Adjusting for body mass substantially decreased the influence of age group and sexual maturity stage on PhA, and particularly the latest remained small at best. Residual analysis showed no spurious correlation between the residuals and body mass, indicating that our model successfully partitioned the influence of body mass on PhA (see Supplementary material).

**Figure 3:**
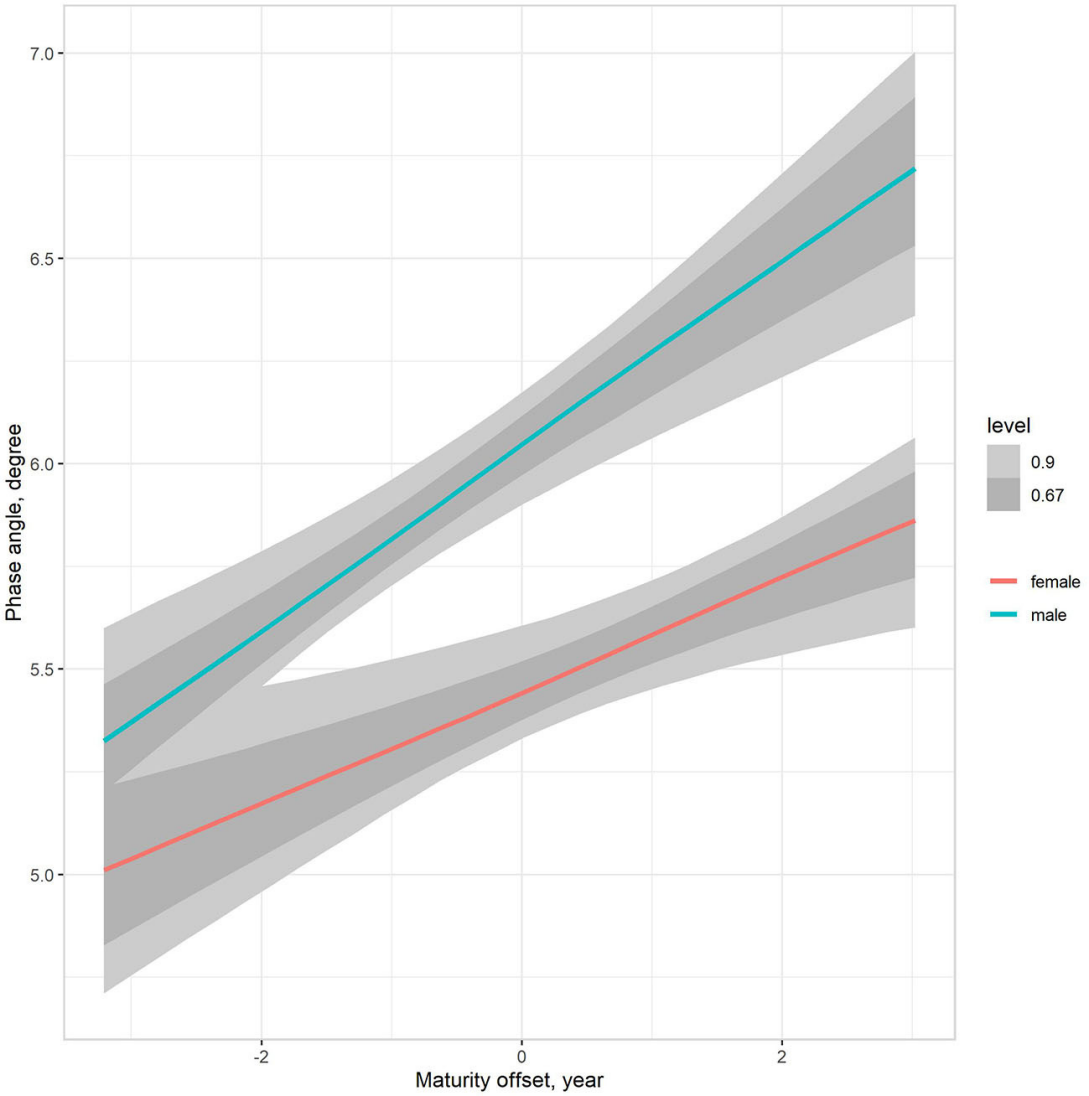
Posterior estimations (67 and 90% CIs) of the association of the phase angle with estimated time before and after the age at PHV (maturity offset) by sex.

**Figure 4:**
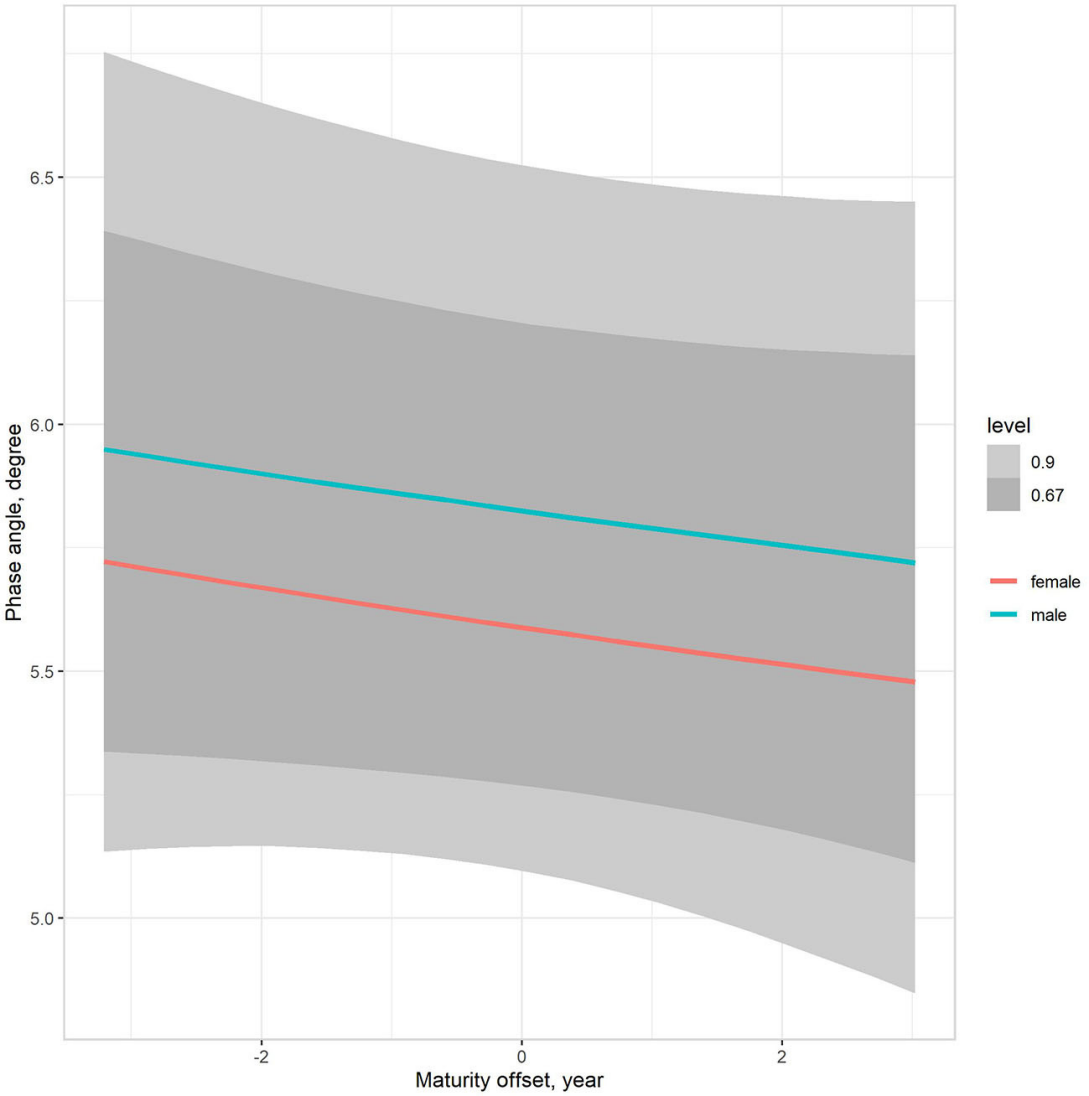
Posterior estimations (67 and 90% CIs) of the association of the phase angle with estimated time before and after the age at PHV (maturity offset) by sex, adjusting for body mass.

The multilevel regression models examining variation in PhA associated with gender, age group, and somatic maturity (model 3) and adjusting for body mass (model 4) are summarized in Table 5. Figure 3 illustrates the relationships between PhA and maturity offset (time before and after predicted age at PHV), contrasting by sex. There was a substantially higher association between PhA and time before and after predicted age at PHV for boys (*r* = 0.31, 90% CI: 0.23 to 0.39) than girls (*r* = 0.2, 90% CI: 0.11 to 0.28). When adjusting for body mass in the model, the association of PhA with the estimated maturity offset became small (*r* = 0.08, 90% CI: 0.02 to 0.15) and similar for girls and boys (Figure 4). The association of body mass with PhA, adjusted for sex and maturity offset, was 0.34 (CI: 0.26 o 0.42). Nevertheless, boys presented higher PhA, adjusted for body mass, than girls when aligned by the time before and after the predicted age at PHV. Residual analysis showed no spurious correlation between the residuals and body mass, indicating that the model successfully partitioned the influence of body mass on PhA (see Supplementary material).

**Table 5:** Multilevel regression models posterior estimations and 90% credible intervals of variation in phase angle by sex, age, group, maturity offset (model 3), and adjusting for body mass (model 4) among adolescents.

	**Model 3** **(variation by sex, age group and maturity offset)**	**Model 4** **(variation by sex, age group and maturity offset, adjusted for body mass)**
Population-level effects (90% credible interval)		
Intercept	0.06 (−1.08 to 1.21)	0.01 (−1.04 to 1.05)
Maturity offset	0.22 (−0.46 to 0.82)	−0.06 (−0.20 to 0.08)
Body mass	-	0.37 (0.28 to 0.45)
Group level estimates (90% credible interval)		
Level 2, standard deviation		
Age group	0.21 (0.01 to 0.72)	0.49 (0.09 to 0.1.25)
Sex		
Intercept	0.90 (0.33 to 1.83)	0.59 (0.09 to 1.51)
Maturity offset (varying slope)	0.42 (0.03 to 1.33)	-
Level 1 standard deviation	0.92 (0.87 to 0.97)	0.88 (0.84 to 0.93)

All outcomes were standardized in the models.

## Discussion

PhA has been considered an important tool for the diagnosis of malnutrition and clinical prognosis, which can be associated with changes in cell membrane integrity, changes in fluid balance, and information on cell health and integrity (i.e., high phase angle values are associated with better permeability of cell membrane and cell function) (7, 40). The present study examined the concurrent influence of sex, age, maturity status, and body size on PhA among Brazilian adolescents. Variation in maturity status significantly influenced the PhA of female and male adolescents aged 10–15 years, adjusting for sex- and age-associated variation. Within each age group, adolescents in more advanced stages of pubic hair, particularly those in late puberty and maturity, had higher values of PhA. Nevertheless, the maturity- and age-associated variation on PhA was significantly accounted for when partitioning for body size. Hence, the influence of body size appears to mediate maturity- and age-associated variation on PhA in adolescents, independent of sex.

The growth characteristics of this sample of Brazilian adolescents were consistent with other reports of healthy young populations (41–43). In addition, the patterns of pubertal growth between individuals and sex-associated variations in the maturity status in the present sample were consistent in longitudinal growth studies in female and male adolescents (20, 21). Also, there is a need to adjust for sex difference in somatic and sexual maturation rates when interpreting maturity-associated variation between girls and boys (44). Hence, age and maturity status must be modeled jointly to analyze sex effects on physiological outcomes. The limitations of the maturity indicators used in this study imply the need for conservative interpretations (45, 46). Nevertheless, the offset equations and the self-examination of pubertal development provide possible options for having a reference of maturity status when only cross-sectional observations are available, assuming their limitations.

Available data consider mainly age-related variation in PhA in adolescents but do not control interindividual differences in the biological maturity status (13, 26, 47–49). Therefore, it appears reasonable to interpret that PhA is likely associated with increased cell mass with age (13). The observations of the presentstudy on Brazilian adolescents indicated substantial variation associated with the stage of pubertal development, adjusting for age. Furthermore, we observed a linear increase in PhA when aligning for the age before and after PHV. Within an age group during adolescence, late-maturing girls and boys appear to have higher values of PhA. Overall, the results suggested an influence of age *per se* on PhA when individual variability is aligned to biological maturity indicators; in the present study, on self-examination of pubertal development and time before or after PHV, it has been noted that PhA values were higher, albeit with small magnitude, in early maturing adolescent football players than in late maturing players, considering the skeletal maturity status using the Tanner-Whithouse-3 method (50). A similar trend has been noted in young football players using somatic maturity status (25, 27), albeit the variation was small. Often, samples of young athletes are relatively homogeneous in maturity status, body size and composition, and sport-specific performance, with a consistent trend of overrepresentation of early maturers (51). Hence, our observations highlight that maturity-associated variation in PhA among non-athletic adolescents is likely substantial and must be accounted for when interpreting PhA.

Biological maturation likely influences PhA through associated variation in somatic features, including size *per se* and lean and fat mass. Pubertal growth is marked by many neuroendocrine changes that mediate changes in size, physique and body composition, and various body systems (45). The process of pubertal growth and maturation, i.e., progress toward the mature state, is related and appears to influence PhA. In particular, our observations suggest that maturity-associated variations in body size and composition, considering body mass as a surrogate of body size and composition, likely explain a substantial portion of age- and maturity-related variation in PhA. Note that the interpretations are similar when considering stature as a surrogate of body size (results available as Supplementary material). In particular, this trend may be related to adolescent growth spurts in the body, and free-fat mass since maximal growth in body and muscle mass occurs after PHV (22).

Our observations showed a clear trend of sex variation, adjusting for age and maturity status. When aligning PhA by age before and after PHV, we observed a distinct increase in trends in PhA, with girls having a lower increase than boys. Nevertheless, the observed sex-related variation in PhA during adolescence became similar between girls and boys when body mass was adjusted in the models. Lean mass is largely constituted of body water (52) and is an excellent conductor of electricity, offering low resistance to the passage of electric current (3, 53). Hence, the low resistance values contribute directly to higher phase angle values (2). Sex dimorphic growth and development are most pronounced during adolescence (54). Girls usually begin adolescent growth before boys and progress at a faster rate than boys (44). Moreover, body fat levels rise substantially in girls during adolescence. Body fat distribution is mainly determined by sex steroids, with increased body fat in girls' subcutaneous, gluteal, and femoral regions (54). The relative contribution of lean mass to total body mass usually declines once consideration is given to the relative contribution of fat mass (22, 54). Furthermore, lean tissue hydration values tend to decline with age in girls, specifically with decreases in water content and increases in density with increasing age. Hence, sex-associated variation in PhA between girls and boys should be expected.

The adequacy and appropriateness of the adopted multilevel regression models were demonstrated by the near-zero relationships between the predicted PhA outputs and body mass and the examination of residuals, which showed a normal distribution (see Supplementary material). Hence, this study illustrates an approach to dealing with concurrent influences of sex, age, maturity status, and body size on physiological outcomes in adolescents. Data and codes for replication of our models are available at https://osf.io/j2yez/.

Our analysis did not account for body composition differences between the adolescents (e.g., fat mass and lean mass). Furthermore, although we took advantage of Bayesian inference in our models, the sample was from the same region within a cross-sectional design, limiting generalizations. Therefore, future studies should adjust PhA considering biological, contextual, and fitness characteristics based on longitudinal observations.

In summary, our study demonstrated significant interindividual variation in PhA among female and male adolescents. Furthermore, the variability in PhA is related to interindividual variation in sex, age, maturity status, and body size differences. Overall, the present study results highlight the need to account for the transient influence of pubertal growth on body size, shape, and composition and its effect on the interpretation. PhA has been proposed to assess body composition using whole-body BIA that can represent the intracellular/extracellular water ratio (5), providing a meaningful clinical interpretation of cell damage, inflammation, or dehydration during pubertal development. Furthermore, the multilevel regression models incorporating body mass indicate that corresponding changes in overall body mass mainly mediate the influence of the sexual and somatic maturity status on PhA. Therefore, for investigating changes in PhA during adolescence, multilevel modeling with standardized parameters is recommended to normalize data, allowing any disproportionate increase in PhA associated with maturity status, sex and body size to be identified.

## Data availability statement

Publicly available datasets were analyzed in this study. This data can be found at: https://osf.io/j2yzez/.

## Ethics statement

The studies involving human participants were reviewed and approved by the Ethics Committee of the Pontifical Catholic University of Campinas (CAAE: 79625817.6.0000.5481) approved the research. All procedures followed Resolution No.466 of 2012 of the National Health Council of the Ministry of Health of Brazil. Written informed consent to participate in this study was provided by the participants' legal guardian/next of kin.

## Author contributions

AM, HC, and GG-J: conceptualization. RQ, AL, and HC: data curation. AM and HC: formal analysis and writing-original draft. GG-J: project administration. AM and GF: investigation. AM, RQ, AL, GF, HC, and GG-J: writing-review and editing. All authors contributed to the article and approved the submitted version.

## Funding

RQ was supported by grants from the Coordenação de Aperfeiçoamento de Pessoal de Nível Superior – CAPES (finance code 001). The study was partially supported by the Fundação de Amparo à Pesquisa do Estado de São Paulo (São Paulo Research Foundation).

## Conflict of interest

The authors declare that the research was conducted in the absence of any commercial or financial relationships that could be construed as a potential conflict of interest.

## Publisher's note

All claims expressed in this article are solely those of the authors and do not necessarily represent those of their affiliated organizations, or those of the publisher, the editors and the reviewers. Any product that may be evaluated in this article, or claim that may be made by its manufacturer, is not guaranteed or endorsed by the publisher.
