# Supplementary material for: Age-, sex-, and maturity-associated variation in the phase angle after adjusting for size in adolescents
Source: Front Nutr. 2022 Aug 1;9:939714. doi: 10.3389/fnut.2022.939714 (PMC9376599; doi:10.3389/fnut.2022.939714)
Supplement: Supplementary file 1 [file Data_Sheet_1.PDF]

Supplementary material for the manuscript
"Age-, sex-, and maturity-associated variation in phase angle after adjusting for size in adolescents"

Anderson M. de Moraes, Ricardo T. Quinaud, Giovana O. C. Ferreira, Ahlan B. Lima, Gil Guerra-Júnior, Humberto M. Carvalho

We present as supplentary material the residual plots of the models including body mass as a predictor. In both Supplementary Figure 1 and Supplementary Figure 2, the multilevel regression models with standardized paramenters were successful partitioning the influence of body. Both plots show that the distribution of the residuals was normaly distributed, and there was no association between the residuals and body mass, implying that the models estimates were independent from body mass.

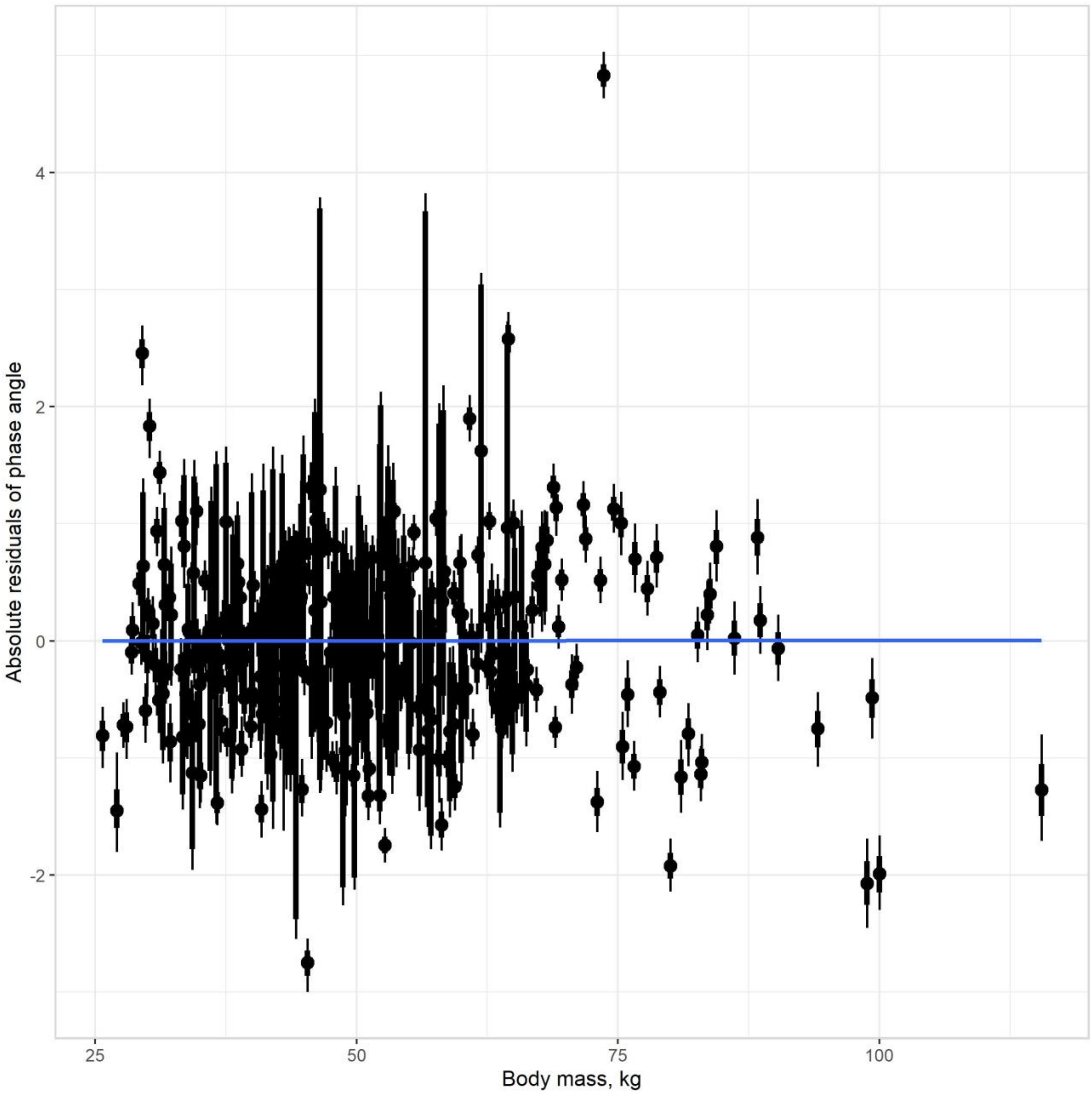

Supplementaty Figure 1. Distribution of residuals against body mass of the model

$$y_i = \beta_0 + \beta_1^{body\ mass} + \alpha_{a[i]}^{age\ group} + \alpha_{s[i]}^{sex} + \alpha_{m[i]}^{maturity\ status} + \epsilon_i$$

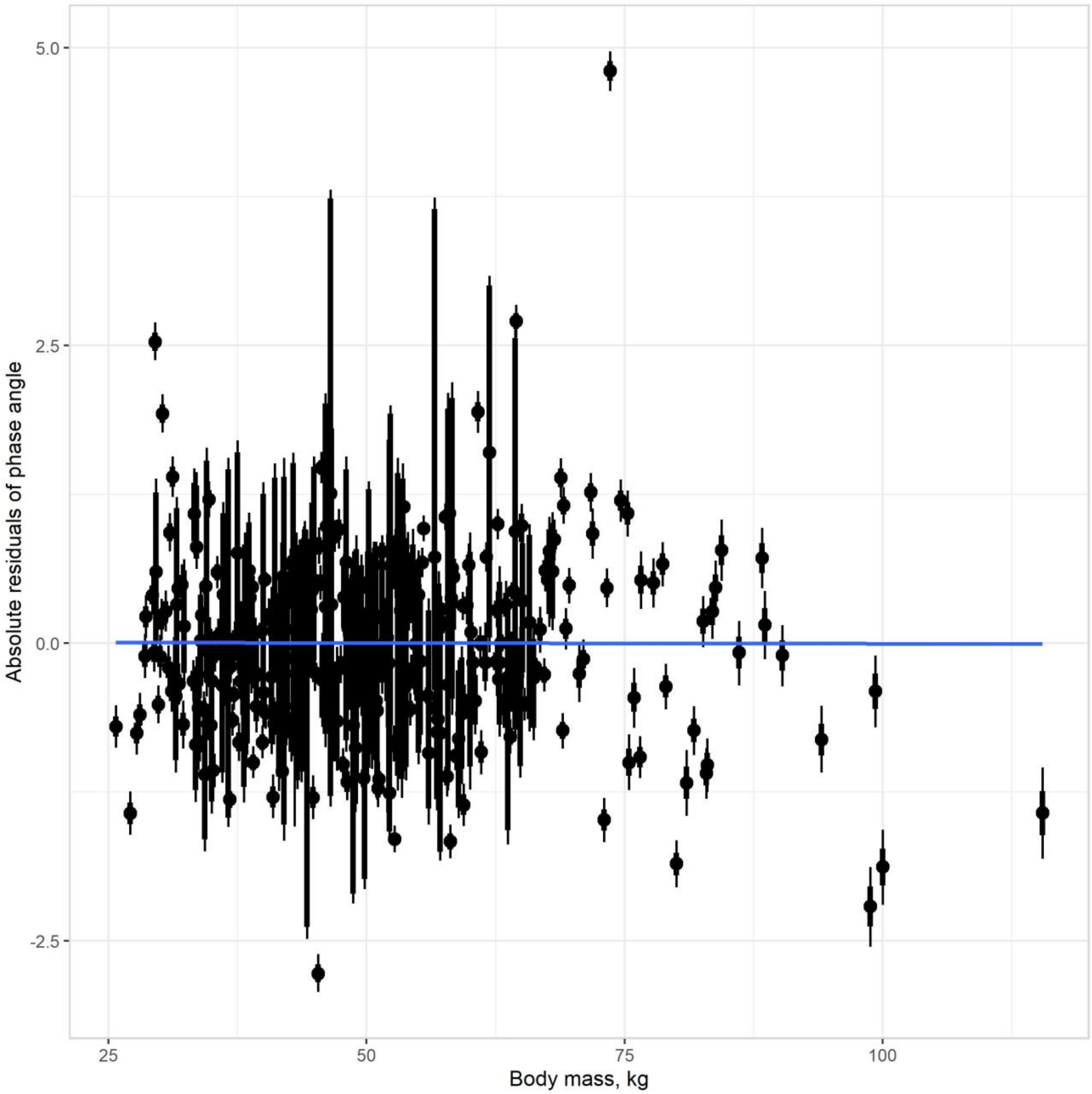

Supplementaty Figure 2. Distribution of residuals against body mass of the model

$$y_i = \beta_0 + \beta_1^{maturity\ offset} + \beta_2^{body\ mass} + \alpha_{s[i]}^{sex} + \epsilon_i$$
