# Supplementary figures and images for: Age-, sex-, and maturity-associated variation in the phase angle after adjusting for size in adolescents
Source: Front Nutr. 2022 Aug 1;9:939714. doi: 10.3389/fnut.2022.939714 (PMC9376599; doi:10.3389/fnut.2022.939714)

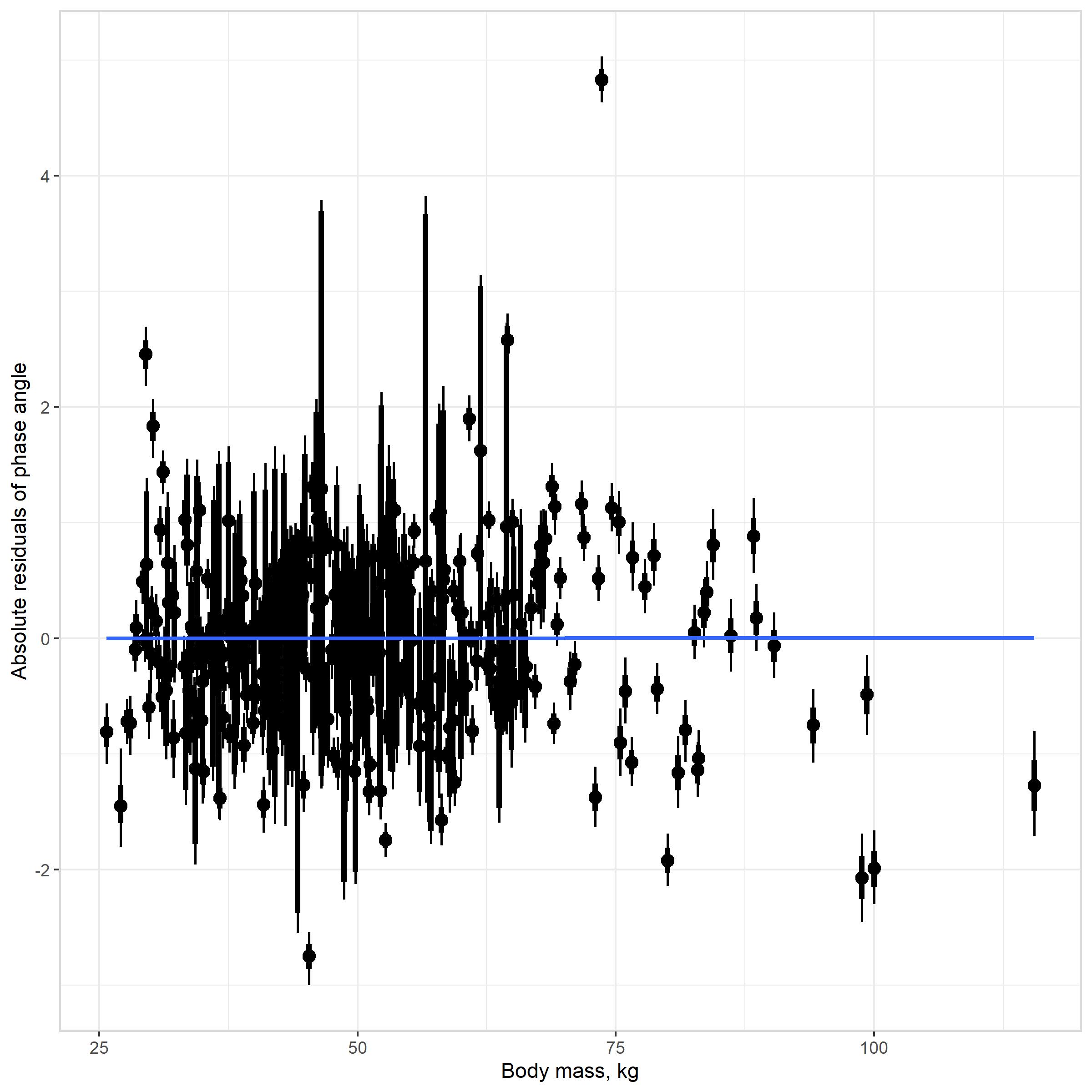

Supplement: Supplementary file 2 [file Image_1.JPEG]

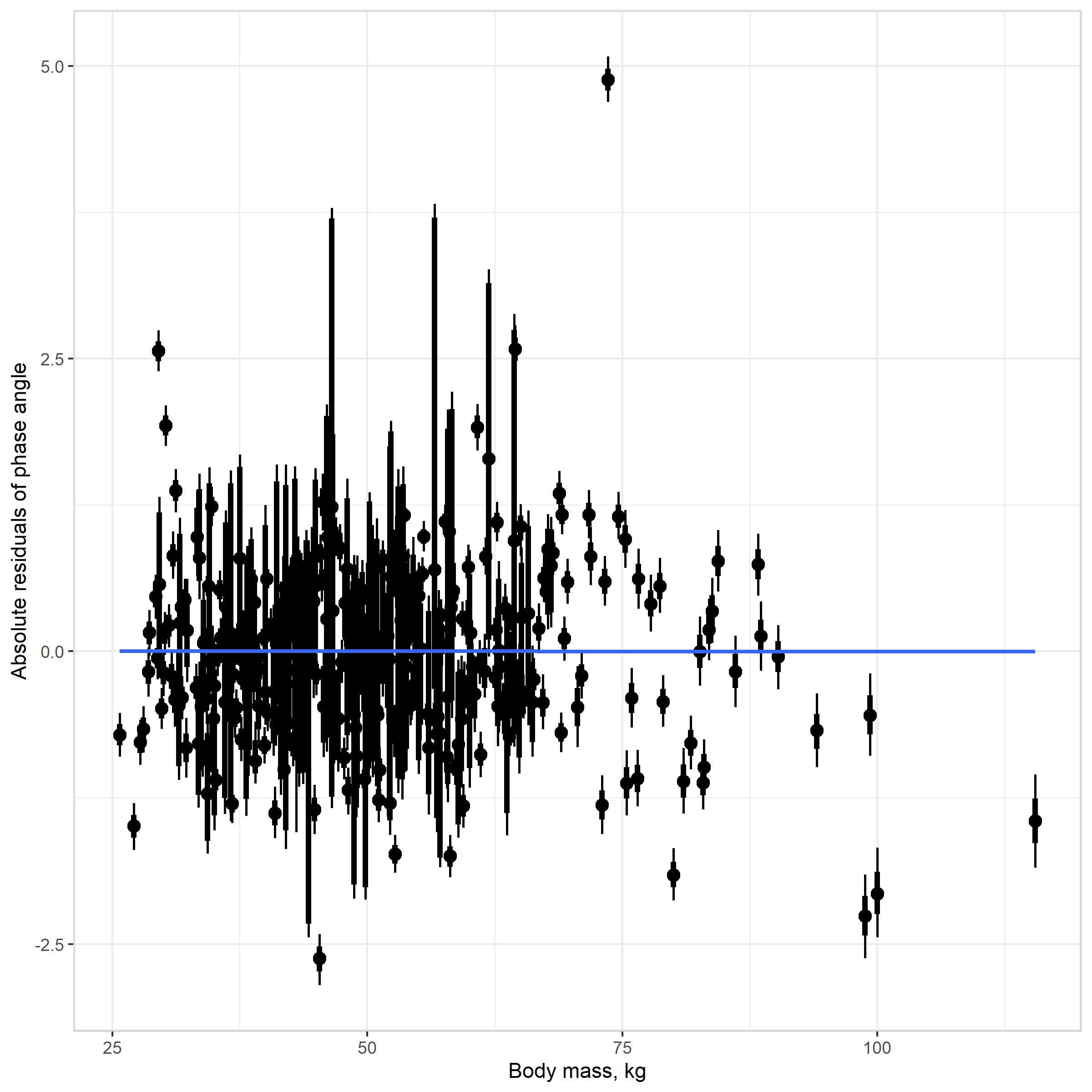

Supplement: Supplementary file 3 [file Image_2.JPEG]

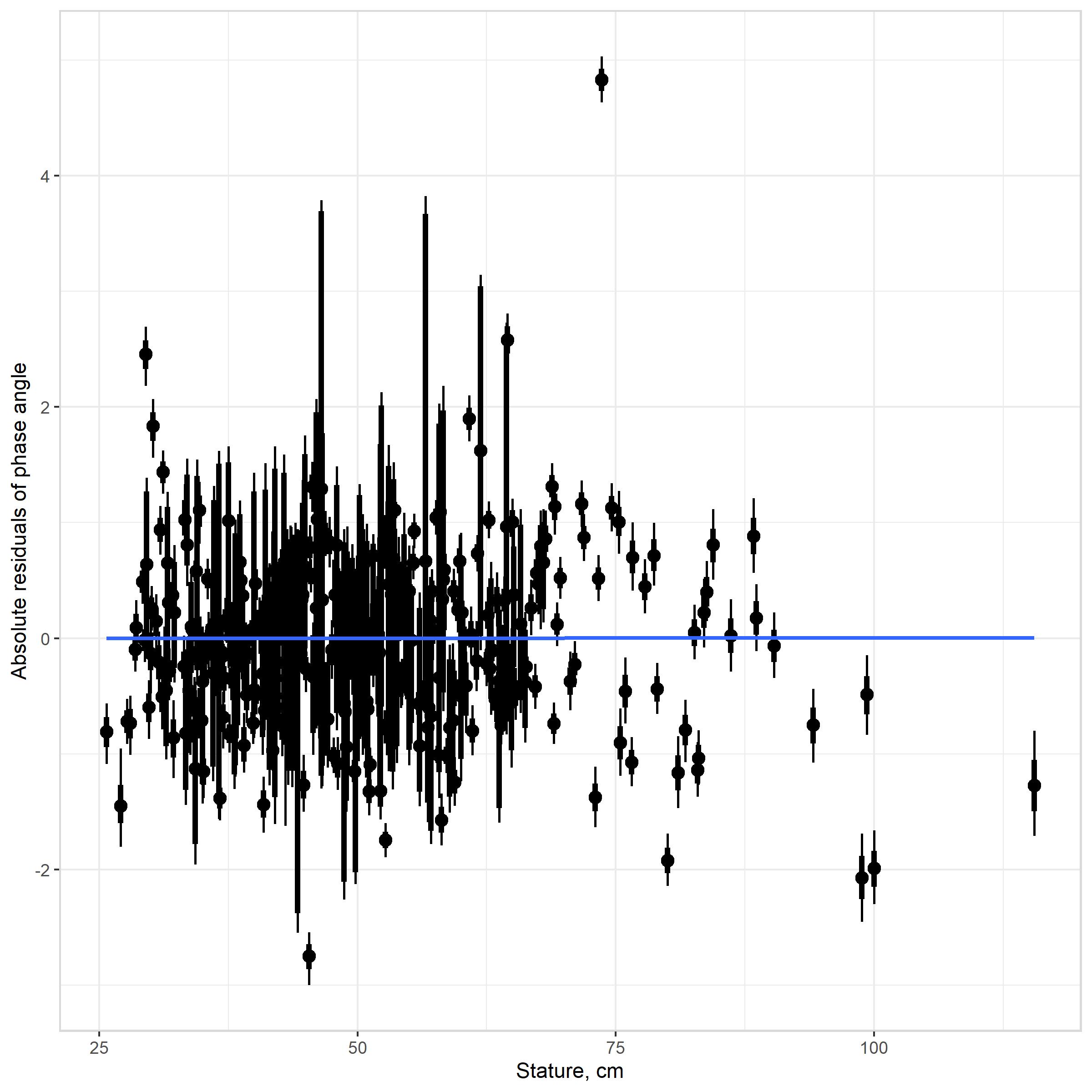

Supplement: Supplementary file 4 [file Image_3.JPEG]

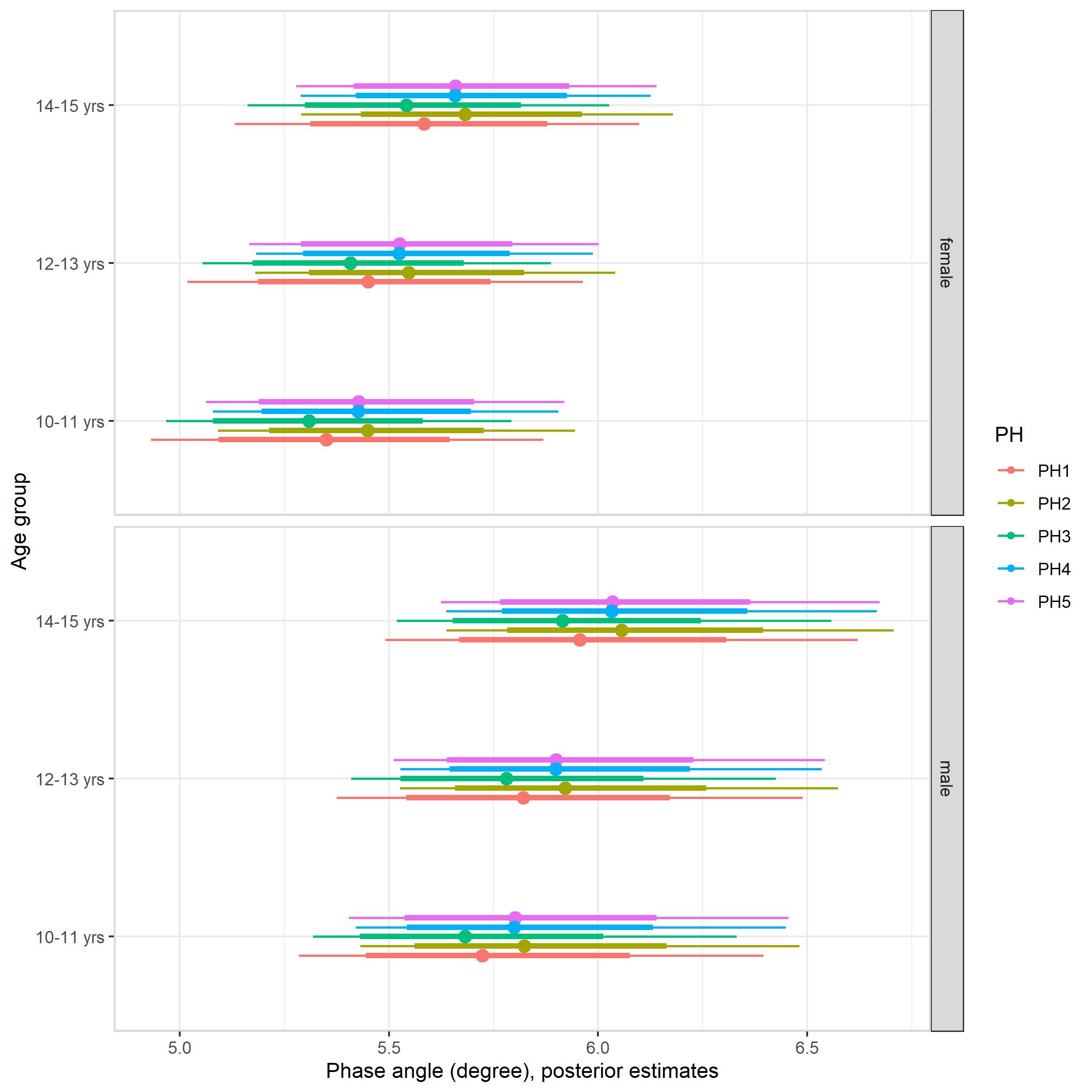

Supplement: Supplementary file 5 [file Image_4.JPEG]

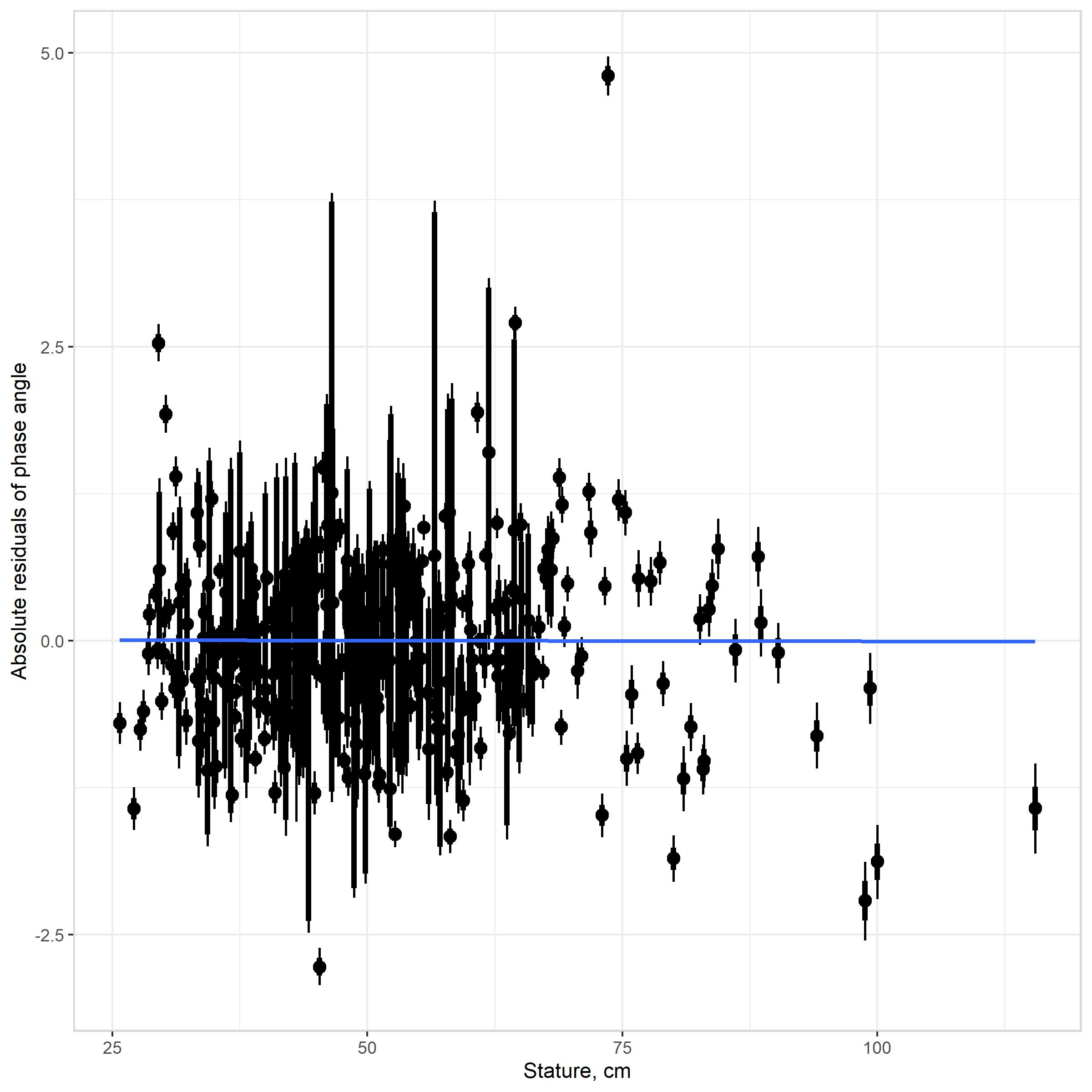

Supplement: Supplementary file 6 [file Image_5.JPEG]

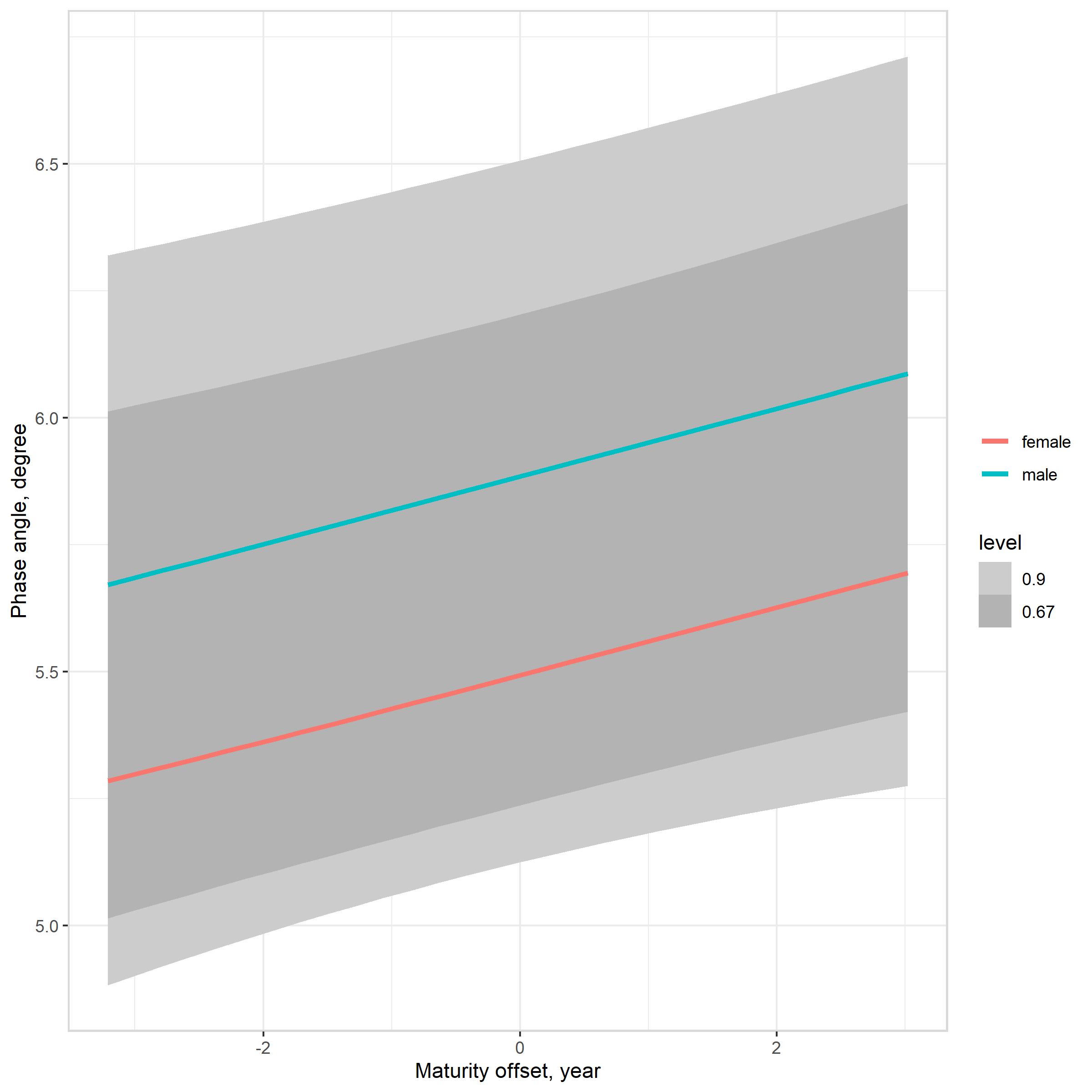

Supplement: Supplementary file 7 [file Image_6.JPEG]
